# Colonization resistance is dispensable for segregation of oral and gut microbiota

**DOI:** 10.1186/s12920-023-01449-3

**Published:** 2023-02-22

**Authors:** Armin Rashidi, Motoko Koyama, Neelendu Dey, Jeffrey S. McLean, Geoffrey R. Hill

**Affiliations:** 1 Fred Hutchinson Cancer Center, 1100 Fairview Ave N, D1-100, Seattle, WA 98109 USA; 2 Division of Oncology, Department of Medicine, University of Washington, Seattle, WA USA; 3 Division of Gastroenterology, Department of Medicine, University of Washington, Seattle, WA USA; 4 School of Dentistry, University of Washington, Seattle, WA USA

**Keywords:** Colonization resistance, Gut microbiota, Oral microbiota

## Abstract

**Background:**

The oral and colonic microbiota are distinct in healthy individuals. However, this distinction is diminished in common diseases such as colon cancer and inflammatory bowel disease, suggesting a potential pathogenic role for oral bacteria when ectopically colonized in the gut. A key mechanism for the segregation of oral and colonic microbiota niches is thought to be microbiota-mediated colonization resistance whereby the commensal gut microbiota outcompete and eliminate the ingested oral bacteria.

**Methods:**

We tested this theory by analyzing exact amplicon sequence variants generated from concurrent fecal and oral samples from healthy volunteers exposed to a brief course of a single antibiotic (cohort 1), acute leukemia patients (cohort 2), and stem cell transplant recipients (cohort 3). Cohorts 2 and 3 represent extreme clinical scenarios with respect to antibiotic pressure and severity of gut microbiota injury.

**Results:**

While mild antibiotic exposure in cohort 1 was not sufficient for colonization of any oral bacteria in the gut, even with extreme antibiotic pressure and severe gut microbiota disruptions in cohorts 2 and 3, only one oral species in each cohort colonized the gut.

**Conclusions:**

Colonization resistance is dispensable for segregation of oral and colonic microbiota in humans. This finding implies that the presence of oral bacteria in the distal gut in diseases such as colon cancer and inflammatory bowel disease is not driven by impaired colonization resistance.

**Supplementary Information:**

The online version contains supplementary material available at 10.1186/s12920-023-01449-3.

## Background

Despite ingesting ~ 10^11^ bacteria per day via saliva, healthy individuals maintain distinct oral and colonic microbiota [1–3]. This contrasts with the small intestinal microbiota which, based on the limited available data, has a substantial overlap with the oral microbiota [4]. In human oral microbiota-associated mouse models, where oral microbiota from humans is transplanted to germ-free mice, the success of oral microbiota in colonizing the gut declines from the more proximal to the more distal sites of the gastrointestinal tract, indicating a more powerful barrier separating the oral microbiota from colonic than small intestinal microbiota [5]. Cumulative evidence indicates a breakdown of the oral/colonic segregation in common diseases such as inflammatory bowel disease (IBD), colon cancer, and rheumatoid arthritis [6–9], suggesting a pathogenic role for oral bacteria ectopically colonized in the distal gut.

Gnotobiotic murine experiments suggest that microbiota-mediated colonization resistance, the process by which the commensal colonic microbiota resist invasion by extra-intestinal microbes, is a key mechanism for the separation of the oral and colonic niches [5, 10]. However, whether this is a critical, indispensable mechanism in humans is unknown. The physicochemical characteristics of the colon (e.g*.* low oxygen pressure, toxins present in fecal content) and multiple antimicrobial mechanisms between the mouth and colon (e.g*.* gastric acid, bile salts, mucosal immunoglobulins, antimicrobial peptides) may create a sufficiently powerful barrier against ectopic colonization, rendering microbiota-mediated colonization resistance dispensable. One of the best examples for how physicochemical properties of the local habitat rather than the host may be the primary determinant of microbiota composition is the fastidious clade *saccharibacteria* (TM7). Dental plaque TM7 resembles TM7 species from environmental non-host habitats more than tongue- and gut-associated TM7, suggesting that host regulation of dental plaque TM7 is weak [11].

To address this knowledge gap, we studied exact amplicon sequence variants (ASVs) generated from 440 pairs of concurrently collected fecal and oral samples from 3 cohorts: (i) healthy individuals exposed to a brief course of a single antibiotic, (ii) patients with acute leukemia receiving chemotherapy, and (iii) allogeneic stem cell transplant recipients. Cohorts 2 and 3 received multiple broad-spectrum antibiotics for several weeks and experienced severe injury to their gut microbiota. We reasoned that if gut colonization resistance played a critical, indispensable role in oral/gut microbiota segregation, the oral and gut microbiota would coalesce in cohorts 2 and 3, and much more prominently so than in cohort 1.

## Methods

We performed a secondary analysis of data from 3 previously published cohorts with concurrently collected stool and oral samples. Cohort 1 [12] included 43 healthy adults who received 5–10 days of a single oral antibiotic (ciprofloxacin ×10 days, clindamycin ×10 days, amoxicillin ×7 days, or minocycline ×5 days). We used data from saliva and stool samples collected immediately after and 1 month after completing the antibiotic course. Cohort 2 included 39 adults with acute myeloid leukemia receiving chemotherapy [13]. We used data from saliva and stool samples collected longitudinally between admission to the hospital for chemotherapy and 1 month after starting chemotherapy. Cohort 3 [14] included 29 children undergoing allogeneic stem cell transplantation. We used data from oral swab and stool samples collected longitudinally between transplant referral and 1 month after transplant. The period chosen for cohorts 2 and 3 represents the interval with the highest antibiotic pressure and most severe gut microbiota disruptions. Samples in these two cohorts were collected approximately every 4 and 7 days, respectively. Subject characteristics and details of sample collection and sequencing in each cohort are available in their corresponding previous publications [12–14].

The sequenced region of the 16S rRNA gene was V5-V7 (Roche 454 pyrosequencing) in cohort 1, V4 (Illumina) in cohort 2, and V3-V4 (Illumina) in cohort 3. In each cohort, ASVs were inferred using DADA2 and taxonomically assigned using the SILVA reference database (version 138.1). The truncation thresholds used in dada2 were 300 bp for cohort 1, 150 (forward) and 130 (reverse) bp for cohort 2, and 280 (forward) and 200 (reverse) bp for cohort 3. Other quality filtration parameters were maxEE = 2 (maximum number of expected errors allowed in a read), and truncQ = 2 (reads truncated at the first instance of a quality score ≤ 2). The DADA2 setting for pooling in the inference step was selected to increase sensitivity in detecting rare variants. Chimeras were identified by sample and removed from each dataset (over all sequencing runs) based on a consensus decision (removeBimeraDenovo() function, method “consensus”).

Pairing of oral and stool samples was done such that each pair included an oral and a stool sample from the same patient at the same timepoint. We measured niche-niche overlap using the Jaccard index [15], defined as the size of the intersection divided by the size of the union of the two samples. Presence/absence data are used to determine this index. Jaccard distance, calculated by subtracting the index from 1, indicates the extent of separation of the two samples. All *p* values were corrected for multiple testing using the Benjamini–Hochberg method [16] and presented as* q* values. We used R 4.2.0 (R Foundation for Statistical Computing, Vienna, Austria) for all analyses. The R script is provided in Additional file 1. The vegan package version 2.6.2 was used to estimate Shannon’s index and Aitchison’s distance (using centered log-ratio ASV abundances) for alpha and beta diversity, respectively. The same package was used to calculate Jaccard distances. dada2 version 1.25.0 was used for ASV inference. The ASV tables for the 3 cohorts are provided in Additional file 2, Additional file 3, and Additional file 4.

The analysis was performed in 3 steps. First, we identified all ASVs overlapping in at least 10% of the fecal/oral sample pairs in each cohort. Next, we used the observed prevalence of each of these ASV in samples of each type in each cohort to calculate the probability of finding the same ASV in both samples of a pair without a need to assume a connection between the two (*i.e.,* random scenario). For each ASV yielded by the first step, an exact binomial test was applied to test the probability of success in a Bernoulli experiment where the expected probability of success was defined by the ASV’s observed prevalence in samples of type 1 multiplied by its observed prevalence in samples of type 2, and the observed number of successes was defined as the number of sample pairs including the ASV in both types. Finally, we argued that bacteria of oral origin, including those that ectopically colonize the gut, are expected to have much higher relative abundances in the mouth than colon. Therefore, we selected the subset of ASVs from step 2 that were more abundant in the mouth than colon.

## Results

**Fig. 1:**
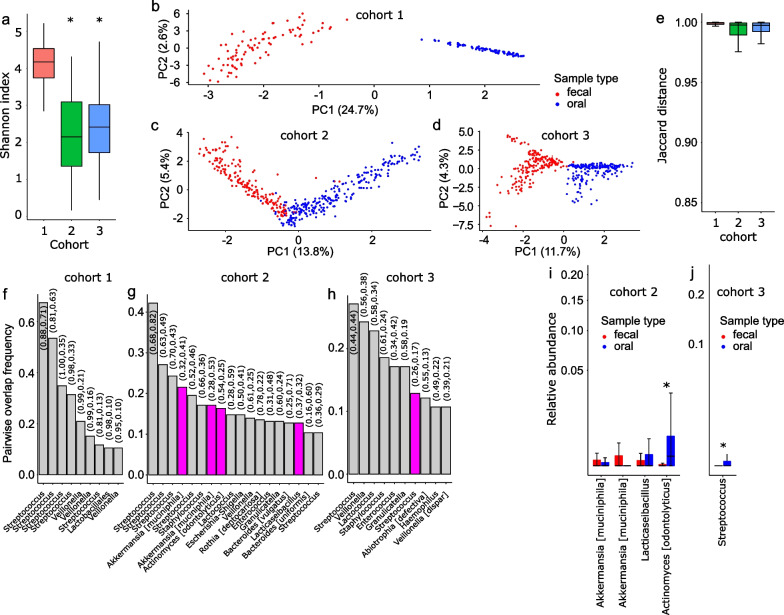
Segregation of oral and colonic microbiota after antibiotic use. Exact short amplicon sequence variants from paired stool and oral samples from 3 cohorts of individuals exposed to antibiotics were analyzed. These cohorts included: (i) 43 healthy adults who received 5–10 days of a single oral antibiotic, (ii) 39 patients with acute leukemia, and (iii) 29 allogeneic stem cell transplant recipients. Cohorts 2 and 3 were exposed to multiple antibiotics for several weeks. **a** Alpha diversity as measured by Shannon index in each cohort. The box boundaries indicate the interquartile range, the horizontal line is the median, and the vertical line shows the range. ^*^q < 0.05 compared to cohort 1. **b**–**d** Principal component analysis using centered log-ratio ASV abundances (Aitchison’s distance). Each point shows a sample. Numbers in square brackets indicate the fraction of total microbiota variation explained by the corresponding axis. **e** Jaccard distance between stool and oral samples of each pair. **f**–**h** Taxa with > 10% pairwise overlap frequency between oral and stool samples. The y-axis shows the observed frequency of overlap. The x-axis shows the deepest level of taxonomy for each ASV. Genera that appear in duplicate represent different ASVs. Taxa shown at species level [in square brackets] represent ASVs that could be unambiguously assigned at that level. Numbers above each bar indicate the observed prevalence of the corresponding ASV in oral (first number) and stool (second number) samples. Magenta bars indicate taxa whose observed frequency of overlap was significantly (*q* < 0.05) higher than expected from their observed prevalence in oral and stool samples. **i**, **j** Relative abundance of significant taxa in panels **f**–**h** in stool versus oral samples. Asterisks indicate taxa with a significantly higher relative abundance in oral samples (*q* < 0.05)

In contrast to cohort 1 who received a brief course of a single antibiotic, cohorts 2 and 3 experienced heavy antibiotic exposure to prevent and treat infections. Few clinical settings are associated with such an extreme antibiotic pressure. Some of the antibiotics used commonly in these cohorts are among the most powerful causes of broad-spectrum injury to the commensal gut microbiota [17]. As an example, 44% of patients in cohort 2 and 97% of those in cohort 3 received carbapenems, a group of strong anti-anaerobic antibiotics that are highly detrimental to the microbiota. Severe injury to the gut microbiota in these cohorts was apparent from their markedly lower alpha diversity compared to cohort 1 (*p* < 10^–15^ from a Kruskal–Wallis test; Fig. 1a). Specific compositional changes in the microbiota in each cohort have been reported in detail [12–14].

After excluding 2 samples from cohort 2 due to low depth (< 1000 reads), we analyzed a total of 440 sample pairs (cohort 1: 86; cohort 2: 214; cohort 3: 140). Median sample depth in cohorts 1–3 was 9664, 20,485, and 37,465 reads, respectively. The number of unique ASVs inferred from these cohorts was 5,302, 6,519, and 2,555, respectively. In principal components analysis using centered log-ratio ASV abundances, the two niches in all cohorts distinctly separated along the first axis (Fig. 1b–d), indicating highly different compositions. The median Jaccard distance between fecal/oral sample pairs in cohorts 1–3 was 0.999, 0.997, and 0.997, respectively, indicating little overlap between the two niches in all cohorts (Fig. 1e). When this analysis was repeated for each sample type longitudinally on a within-individual basis, the values were 0.71 (fecal) and 0.67 (oral) for cohort 1, 0.81 (fecal) and 0.84 (oral) for cohort 2, and 0.88 (fecal) and 0.89 (oral) for cohort 3. This comparison indicated major alterations in both oral and gut microbiota over time, more in cohorts 2 and 3 than in cohort 1, as expected from the extent of their antibiotic exposure. Also, these distances are smaller than those obtained from paired-sample analysis, indicating more similarity between samples of each type for an individual than between oral and fecal sample pairs.

The first step of analysis yielded 9, 18, and 10 ASVs in cohorts 1–3, respectively, that overlapped in at least 10% of the fecal/oral sample pairs (Fig. 1f–h). The most frequent taxon corresponding to these ASVs was *Streptococcus* (5, 5, and 2 ASVs in cohorts 1–3, respectively). *Veillonella* was the only other taxon that was present on the list of overlapping ASVs in all cohorts. In step 2 of analysis, the observed overlap was significantly (*q* < 0.05) more frequent than expected from a random scenario for none of the 9, 4 of the 18, and 1 of the 10 overlapping ASVs in cohorts 1–3, respectively (magenta bars and observed frequencies in Fig. 1f–h). Thus, we found no evidence for ectopic colonization in cohort 1. The 4 ASVs emerging from this step in cohort 2 were mapped to *Akkermansia muciniphila* (2 ASVs), *Lacticaseibacillus* (1 ASV), and *Actinomyces odontolyticus* (1 ASV). The only ASV emerging from this step in cohort 3 was *Streptococcus*. In the third and final step, only 1 of the remaining 4 ASVs on the list for cohort 2 was significantly more abundant in the oral sample than the stool sample of the same pair (paired Wilcoxon’s *q* = 2.3 × 10^–12^) (Fig. 1i). This ASV was mapped to *Actinomyces odontolyticus*, a predominant species in supragingival and subgingival plaques [18]. In cohort 3, the only remaining ASV on the list (mapped to *Streptococcus*) was significantly more abundant in the oral sample than the stool sample of the same pair (*q* = 0.014) (Fig. 1j). Therefore, minimal ectopic colonization was present in cohorts 2 and 3 despite high antibiotic pressure.

## Discussion

Determinants of microbial colonization and niche partitioning within the host are a major question in microbiology and microbial ecology. Physicochemical and biological properties of different habitats and microbial evolution and adaptation to those properties and host factors together determine microbiota composition in each niche [19]. The composition of the gut microbiota varies along the length of the gut. Factors that determine niche specificity of the microbiota include epithelial cell types and surfaces, mucus thickness, motility and contractility, pH, oxygen tension, and flow rate that vary along the gastrointestinal tract. For example, direct exposure of the oral cavity to external oxygen favors aerobic and facultative anaerobic bacteria, while the deeply hypoxic colonic lumen favors obligate anaerobes. Saliva impacts the oral microbiota by releasing antimicrobial peptides, nutrients (via digestive enzymes), and mucin [20]. The esophageal microbiota resembles the oral microbiota and is heavily influenced by diet [21]. The gastric microbiota is less abundant than more distal sites due to its high acidity, mucosal thickness, and peristalsis [22]. The small intestine also has a lower microbial load than colon, largely due to its rapid transit time which opposes stable colonization, but also due to antimicrobial compounds such as bile acids and digestive enzymes [23]. Moving from the more proximal segments of the small intestine (e.g. duodenum) toward the colon, the oxygen tension progressively declines [24], and this mirrors the relative abundance of commensal anaerobic bacteria [25]. The microbiota in the most distal parts of the small intestine is influenced by the relatively thin mucus layer and high abundance of antimicrobial peptides made by Paneth cells [26]. The host and environmental factors experienced by the colonic microbiota are unique and consist of a thick mucus layer, slow transit time, and deep luminal hypoxia.

Using exact sequence variants, oligotypes, and metatranscriptomics, we and others have demonstrated that the oral and colonic microbiota are distinct in healthy adults [1–3]. While this strict niche partitioning indicates the physiological state with intact microbiota-host homeostasis, several pathologic states have been associated with a breakdown of the oral/colonic microbiota segregation. Several taxa associated with putative oral pathobionts are enriched in the gut of patients with IBD. These include *Prevotella*, Porphyromonadaceae, *Neisseria*, *Veillonella*, and *Atopobium* [6]. In patients with colorectal cancer, *Fusobacterium nucleatum* is the hallmark species overlapping between oral and colonic (and colon cancer tissue) microbiota [7, 27]. Similarly, *Lactobacillus salivarius* is present in dental, salivary, and fecal microbiota of patients with rheumatoid arthritis [9]. The question that inspired the present work was: what does it take for the oral/colonic microbiota barrier to break down? Specifically, we evaluated whether extreme antibiotic pressure (cohorts 2 and 3 as opposed to cohort 1), expected to cause major disruptions to the indigenous commensal gut microbiota and colonization resistance, is sufficient for microbiota coalescence. Although colonization resistance is known to contribute to the protection of the intestinal niche against extrinsic pathogens (e.g. enteric pathogens such as *Campylobacter jejuni*) in humans [28], whether this contribution is essential or dispensable by other physiological mechanisms in unknown. In gnotobiotic mice receiving human oral microbiota transplants, several taxa were eliminated by the distal gut where a low-diversity community was established consisting mostly of *Streptococcus*, *Veillonella*, *Haemophilus*, *Fusobacterium*, *Trichococcus*, and *Bacteroides* [5].

Although ASVs are resolved at the level of single nucleotide differences and identify taxa at a higher resolution than operational taxonomic units, because the analyzed data were short amplicon sequences, more than one strain or species of bacteria may correspond to the same ASV. Therefore, even the infinitesimal coalescence of the two microbiota in cohorts 2 and 3 may be an overestimate. One potential limitation of the present analysis is related to antibiotic-related changes in the oral microbiota, which although less significant than those in the gut microbiota, were nonetheless present [12–14]. We cannot eliminate the possibility that specific members of the oral microbiota with potential for ectopic colonization were removed by antibiotics. Such antibiotic-sensitive members of the oral microbiota could have colonized the gut with disrupted indigenous microbiota in these patients. Specific oral streptococci and *Porphyromonas gingivalis*, for example, can colonize the distal gut in murine experiments [29–31]. This scenario would be difficult to ascertain in the patients analyzed herein because with a few exceptions (e.g. oral vancomycin), antibiotics administered orally or intravenously reach the oral cavity.

It is possible that specific colonic niches are needed for successful colonization of oral bacteria. The antibiotics used in the 3 cohorts analyzed here may not have been able to free the right niches for oral microbiota colonization. This is likely more relevant to individuals in cohort 1 because they received a single antibiotic. In cohort 1, some individuals received clindamycin (with anti-anaerobic activity), but the other antibiotic classes had limited to no anti-anaerobic activity. In contrast, cohorts 2 and 3 frequently received strong anti-anaerobic antibiotics such as carbapenems and piperacillin-tazobactam. Vancomycin (oral or intravenous) was another antibiotic frequently used in cohorts 2 and 3. Oral vancomycin is unique in that due to lack of absorption, its effect on the microbiota is limited to the intestines; this includes a large array of anaerobic bacteria [32–34]. Intravenous vancomycin, when used multiple times and for several days, reaches the intestinal lumen and exerts similar effects to those by oral vancomycin. Overall, antibiotic exposure in cohorts 2 and 3 was much more extensive and potentially increased the likelihood of ectopic colonization more than in cohort 1, though the inevitable collateral damage to the oral microbiota was also probably more severe in cohorts 2 and 3, decreasing the pool of oral bacteria that could colonize the distal gut. In addition, damage to the colonic mucosal barrier due to anti-leukemia chemotherapy and transplant conditioning in cohorts 2 and 3 may render the colonic environment less receptive to new microbes coming from the mouth. We cannot exclude the contribution of these factors to the absence of distal gut colonization by oral bacteria in cohorts 2 and 3.

The objective of the present study was not a meta-analysis, thus the cohorts were intentionally selected to be from different settings and even the sequencing methods somewhat differed among the studies. These differences were not detrimental to our analysis because we did not intend to derive an ensemble estimate of association or effect using meta-analytic statistical approaches. Rather, our consistent findings from 3 cohorts despite their demographic and clinical differences and different sequencing methodological details demonstrate that the mechanisms that separate the oral and gut microbiota together form a highly robust barrier that maintains near-complete niche segregation even under extreme antibiotic pressure.

## Conclusions

We demonstrate that gut microbiota-mediated colonization resistance is dispensable for the segregation of gut and oral microbiota. The unique, intrinsic physicochemical properties of each niche likely play the primary role in maintaining a distinctly adapted microbiota while the multitude of antimicrobial barriers along the gastrointestinal tract prevent successful transmission of bacteria from the mouth to colon. Therefore, mechanisms other than diminished colonization resistance drive ectopic colonization of oral bacteria in the distal gut in disease states.

## Supplementary Information

**Additional file 1**. R code.

**Additional file 2**. ASV table for cohort 1.

**Additional file 3**. ASV table for cohort 2.

**Additional file 4**. ASV table for cohort 3.

## Data Availability

Sequencing data from each cohort are publicly available in their corresponding previous publications (References [12–14]). The R code is provided in Additional file 1. The ASV tables for the 3 cohorts are provided in Additional file 2, Additional file 3, and Additional file 4.

## Abbreviations

ASV: Amplicon sequence variant
DADA2: Divisive Amplicon Denoising Algorithm
rRNA: Ribosomal ribonucleic acid
