# Supplementary material for: Colonization resistance is dispensable for segregation of oral and gut microbiota
Source: BMC Med Genomics. 2023 Feb 22;16:31. doi: 10.1186/s12920-023-01449-3 (PMC9948407; doi:10.1186/s12920-023-01449-3)
Supplement: Supplementary file 1 — Additional file 1. R code. [file 12920_2023_1449_MOESM1_ESM.docx]

library(dada2)

library(vegan)

library(microbiome)

library(phyloseq)

##dada2

path <- "path to folder containing fastq files"

fnFs <- sort(list.files(path, pattern=".1.fastq")) ##adjust pattern as needed

fnRs <- sort(list.files(path, pattern=".2.fastq")) ##adjust pattern as needed

sample.names <- sapply(strsplit(basename(fnFs), "[.]"), `[`, 1) ##adjust as needed

filtFs <- file.path(path, "filtered", paste0(sample.names, "_F_filt.fastq.gz"))

filtRs <- file.path(path, "filtered", paste0(sample.names, "_R_filt.fastq.gz"))

names(filtFs) <- sample.names

names(filtRs) <- sample.names

## filter and trim

##replace x and y in the next line with desired truncation lengths for forward and reverse reads

out <- filterAndTrim(fnFs, filtFs, fnRs, filtRs, truncLen=c(x,y),maxN=0,maxEE=2,truncQ=2,rm.phix=TRUE)

## learn error rates

errF <- learnErrors(filtFs)

errR <- learnErrors(filtRs)

## dereplication

derepFs <- derepFastq(filtFs)

derepRs <- derepFastq(filtRs)

names(derepFs) <- sample.names

names(derepRs) <- sample.names

## sample inference

dadaFs <- dada(derepFs, err=errF, pool="pseudo")

dadaRs <- dada(derepRs, err=errF, pool="pseudo")

mergers <- mergePairs(dadaFs, filtFs, dadaRs, filtRs, verbose=TRUE)

## construct sequence table

seqtab <- makeSequenceTable(mergers)

## remove chimeras

seqtab.nochim <- removeBimeraDenovo(seqtab, multithread=FALSE, verbose=TRUE)

## assign taxonomy

taxa <- assignTaxonomy(seqtab.nochim, "path to silva_nr99_v138.1_train_set”)

taxa <- addSpecies(taxa, "path to silva_species_assignment_v138.1”)

samples.out <- rownames(seqtab.nochim)

## make a phyloseq object

samdf<-read.csv("metadata.csv")

samdf<-samdf[match(samples.out, samdf$Sample_ID),]

rownames(samdf) <- samples.out

ps <- phyloseq(otu_table(seqtab.nochim, taxa_are_rows=FALSE), sample_data(samdf), tax_table(taxa))

taxa_names(ps) <- paste0("ASV", seq(ntaxa(ps)))

##Alpha diversity, Shannon index

sample_data(ps)$SDI<-diversity(ps,index='Shannon')

##Beta diversity, Aitchison distance

ps<-microbiome::transform(ps,transform="clr")

ord_clr <- phyloseq::ordinate(ps, "RDA")

sapply(ord_clr$CA$eig[1:5], function(x) x / sum(ord_clr$CA$eig))

clr1 <- ord_clr$CA$eig[1] / sum(ord_clr$CA$eig)

clr2 <- ord_clr$CA$eig[2] / sum(ord_clr$CA$eig)

phyloseq::plot_ordination(ps, ord_clr, type="samples", color="SampleType") + geom_point(size =0.3) +

coord_fixed(clr2 / clr1) + theme_classic()+scale_color_manual(values=c("red","blue"))

##Jaccard distance between paired samples

j_dis<-vector()

for (i in unique(sample_data(ps)$ timepoint)){

ps_t<-subset_samples(ps, timepoint==i)

for (j in unique(sample_data(ps_t)$PatientID)){

ps_tj<-subset_samples(ps_t,PatientID==j)

j_dis<-append(j_dis,vegdist(otu_table(ps_tj),method = "jaccard")[1])

}

}

##Jaccard distance within each sample type (example fecal)

ps_f<-subset_samples(ps,SampleType=="fecal")

j_dis<-vector()

for (j in unique(sample_data(ps_f)$PatientID)){

ps_f_j<-subset_samples(ps_f,PatientID==j)

j_dis<-append(j_dis,c(vegdist(otu_table(ps_f_j),method = "jaccard")))

}

##ASV overlap

prev <- function(x) {sum(x==0)}

ps_f<-subset_samples(ps, SampleType ==" fecal ")

ps_o<-subset_samples(ps, SampleType =="oral")

in_f<-names(which(apply(otu_table(ps_f),2,prev)<nrow(otu_table(ps_f))))

in_o<-names(which(apply(otu_table(ps_o),2,prev)<nrow(otu_table(ps_o))))

overlap<-intersect(in_f,in_o)

ps <- prune_taxa(overlap, ps)

ps<-subset_samples(ps,sample_sums(ps)>0)

ps <- transform_sample_counts(ps, function(x) x / sum(x))

df<-cbind(sample_data(ps),otu_table(ps))

dg<-df[1,x:ncol(df)] ##replace x with the index for the first ASV column

N<-vector()

for(k in unique(sample_data(ps)$timepoint)){

dfT<-subset(df,df$timepoint==k)

N<-append(N,length(unique(dfT$PatientID)))

n1<-vector()

for(i in x:ncol(df)){

n<-0

for(j in unique(dfT$ PatientID)){

dfTpt<-subset(dfT,dfT$ PatientID ==j)

u<-subset(dfTpt,dfTpt$ SampleType =="oral")[,i]

v<-subset(dfTpt,dfTpt$ SampleType =="fecal")[,i]

if(length(u)*length(v)>0){

if(u>0 & v>0){n<-n+1}

}

}

n1<-append(n1,n)

}

dg<-rbind(dg,n1)

}

dg<-dg[-1,]

dg<-dg[,which(colSums(dg)/sum(N)>0.1)]

dg<-colSums(dg)/sum(N)

dg<-as.data.frame(dg)

dg$ASV<-row.names(dg)

##Observed vs. expected frequency of overlap

##Replace y with the number of overlapping ASVs from the last step

##dg is a database, where each row contains data for an ASV and columns are “both” (observed number of sample pairs with the overlapping ASV), “total” (total number of sample pairs), “oral” (observed prevalence of the overlapping ASV among oral samples), and “fecal” (observed prevalence of the overlapping ASV among fecal samples).

p_vector<-vector()

for(i in 1:y){

p_vector<-append(p_vector,binom.test(dg$both[i],dg$total[i],p=dg$oral[i]*dg$fecal[i],alternative ="greater")$p.val)

}

p.adjust(p_vector,method='fdr')
